# CellSigFlow: a flow-based optimization framework for realistic single-cell and spatial inference of intercellular communication

**DOI:** 10.1093/bib/bbaf631.016

**Published:** 2025-12-12

**Authors:** Menghan Wang, Junya Yang, Jiaxing Chen

**Affiliations:** Guangdong Provincial/Zhuhai Key Laboratory IRADS, Beijing Normal-Hong Kong Baptist University, JinTong Road, 519087, Zhuhai, China; Guangdong Provincial/Zhuhai Key Laboratory IRADS, Beijing Normal-Hong Kong Baptist University, JinTong Road, 519087, Zhuhai, China; Guangdong Provincial/Zhuhai Key Laboratory IRADS, Beijing Normal-Hong Kong Baptist University, JinTong Road, 519087, Zhuhai, China

## Abstract

**Background:**

Cell–cell communication (CCC) is fundamental for tissue organization, developmental programs, and disease progression. The advent of single-cell and spatial transcriptomics has made it possible to infer ligand–receptor (L–R) interactions at unprecedented resolution, and numerous computational frameworks have been proposed to integrate gene expression with spatial information to predict putative signaling events.

Early approaches such as CellPhoneDB and CellChat (1) infer L–R interactions primarily at the cluster level. While these methods capture global signaling patterns, they cannot resolve communication at the level of individual cells. Moreover, they typically analyze each L–R pair in isolation, which limits their ability to reveal the complexity of real signaling networks that involve multiple ligands and receptors acting in concert.

Recent tools designed for spatial transcriptomics, including Giotto, NICHES, and COMMOT(2), incorporate neighborhood information or optimal transport to achieve cell-level predictions. Despite these advances, there are still important limitations. Existing methods often overlook competition among receptors and ligands within a cell, and they rarely model the capacity constraints imposed by the ligand or receptor abundance. As a result, predicted communication edges may lack biological realism, showing either spurious absence or unrealistic saturation of signaling. Furthermore, most frameworks remain restricted to single-step L–R pairs, leaving multistep relay cascades largely unexplored.

To address these challenges, we propose a method called CellSigFlow, which formulates CCC as a bounded minimum-cost flow problem. In this framework, each cell is represented as a node and receptor-centric directed edges are constructed, with communication strength modeled as edge flow. Spatial distance and molecular affinity are incorporated into the cost function to penalize long-range interactions and favor biologically plausible binding. By introducing upper bounds proportional to receptor abundance and lower bounds informed by prior knowledge, the model enforces non-zero yet biologically constrained fluxes. This design enables the framework to capture competition among multiple receptors for the same ligand within a cell, resolve signaling at the single-cell level, and naturally integrate multiple L–R pairs to reconstruct complex communication networks beyond isolated interactions.

**Methods:**

According to our method CellSigFlow, we formulate CCC as a bounded minimum-cost flow problem on a spatially constrained graph. Each cell is represented as a node. For a target cell 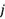, and for each receptor 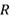 expressed in 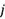, we create a directed edge 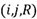 from a source cell 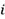 if the spatial distance 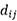 is below a cutoff 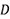, and there exists at least one ligand 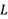 expressed in 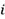 that can bind 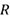 in 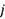. Thus, a pair of cells may be connected by multiple edges only to the extent of distinct receptors on the target; communication strength is modeled as the flow 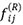 on edge 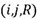.

For each receptor-centric edge, the flow is constrained by a lower bound 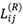 and an upper bound 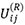:

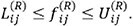

Upper bounds are proportional to receptor abundance in 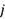 together with the aggregate availability of ligands in 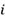 that bind 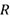, while lower bounds incorporate prior knowledge (e.g., CellPhoneDB, CellChat), ensuring nonzero yet biologically plausible signaling at the receptor level.The complete bounded minimum-cost flow formulation requires that, in addition to satisfying upper and lower capacity constraints, the system obeys flow conservation at each node and minimizes the total transportation cost. The receptor-specific cost on edge is defined as

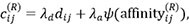

where 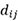 denotes the spatial distance between cells and 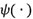 encodes a receptor-centric affinity term that aggregates the contributions of all ligands in 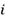 capable of binding receptor 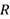 in 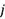. The optimization objective is therefore to minimize the total cost

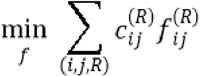

subject to

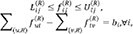

where 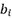 denotes the net supply or demand at node 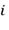 (typically zero). For efficient computation, we transform the above bounded problem into a minimum-cost maximum-flow (MCMF) formulation. Specifically, we define residual variables

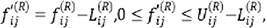

and adjust node demands accordingly:

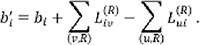

Here, nodes with 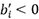 are connected to a super-source, and nodes with 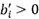 to a super-sink, via auxiliary edges of corresponding capacity and zero cost. A feasible solution exists only if all auxiliary edges are fully saturated. The total minimum cost equals the fixed contribution from all lower bounds plus the minimum cost of the residual flow. The original solution is finally recovered by

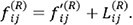

After computing the receptor-centric edge flows 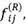 CellSigFlow further refines the signaling resolution to the level of individual L-R. Specifically, for each receptor R in target cell j, the total incoming flow 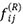 from source cell i is redistributed among all ligands L expressed in i that can bind R. The redistribution weights are determined by a combination of ligand—receptor affinity and ligand gene expression levels, such that

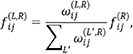

where

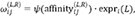

with 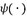 representing the affinity-based weighting function and 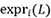 denoting the expression level of ligand 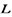 in cell 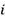. This hierarchical redistribution enables CellSigFlow to derive interpretable single-ligand—single-receptor signaling flows while retaining the global optimization structure of the bounded flow model. This bounded flow formulation naturally introduces a competition mechanism: the capacity of each receptor-centric edge is limited, so if one L-R pair occupies a larger portion of the flow, the available capacity for other potential interactions is reduced. Consequently, interactions with stronger affinity or more favorable spatial positioning will `seize” limited signaling resources, suppressing weaker competitors. Such competition occurs not only among different ligands targeting the same receptor but also in the allocation of signaling from one source cell to multiple neighboring targets. This mechanism effectively reflects the biological resource constraints and exclusivity inherent in molecular and cellular interactions.

**Results:**

**Benchmarking with PDE-based simulations:**

Direct validation of CCC inference methods is limited by the scarcity of ligand-receptor co-localization data. To establish controlled benchmarks, inspired by COMMOT’s simulations, we built a series of partial differential equation (PDE)-based reaction–diffusion models simulating ligand diffusion, production, degradation, and receptor binding dynamics, thereby capturing essential biophysical processes of intercellular signaling. We designed three representative regimes: Type 1, multiple ligands competing for few receptors; Type 2, multiple receptors competing for few ligands; and Type 3, complex interconnected ligand—receptor networks. By varying the numbers and connectivity of ligands and receptors, we generated synthetic networks spanning these scenarios. CellSigFlow was benchmarked against CellChat and COMMOT using multiple evaluation metrics.

The average Spearman correlations (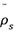) are summarized below:

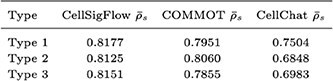

Across all regimes, CellSigFlow achieved the highest agreement with ground-truth communication fields, outperforming both alternatives. These PDE-based simulations demonstrate that CellSigFlow more faithfully captures intercellular signaling as continuous spatial flows.

**Mouse Olfactory Bulb Data Analysis:**

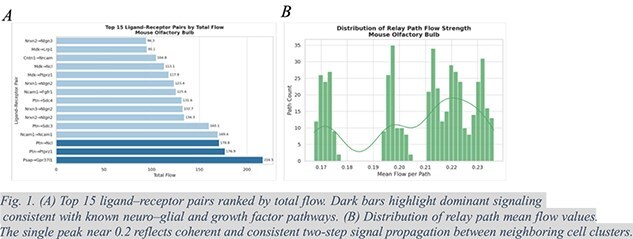

CellSigFlow was applied to the 10x Genomics Visium spatial transcriptomics data of the mouse olfactory bulb, generating a connected communication network of 191,498 directional edges across 1185 spatial spots. The analysis revealed 591 ligand—receptor pairs and 500 two-step relay paths, outlining coherent signaling hierarchies across the tissue. The Psap-Gpr37l1 axis showed the highest cumulative flow (216.5), consistent with astrocyte-to-neuron trophic signaling that supports neuronal survival and glial homeostasis. Ptn-Ptprz1, Ptn-Ncl, and Ptn-Sdc3 formed a pleiotrophin (PTN) module promoting neurite outgrowth and synaptic plasticity, while Ncam1-Ncam1 reflected homophilic adhesion crucial for axon guidance and synapse stabilization. Moderate-flow axes such as Fgfr1-Fgfr1 and Sdc4-Fgfr1 indicated fibroblast growth factor (FGF) signaling involved in structural maintenance. Relay path analysis showed consistent mean flow (0.17–0.24), suggesting coordinated rather than random signal propagation. Notably, cascades such as Ptn → Ptprz1 → Cadm3/Nectin1 → Cadm3 and Psap→Gpr37l1 → Nlgn3/Cadm3 linked growth factor and adhesion signaling, mirroring mechanisms of synaptic maturation. Together, these findings demonstrate that CellSigFlow captures both direct and multi-step communication events underlying neuro–glial interactions and synaptic organization in spatial tissue contexts.

**Conclusion:**

In this work, we introduced CellSigFlow, a novel framework that formulates CCC as a bounded minimum-cost flow problem. By explicitly modeling receptor-level constraints, ligand availability, and spatial context, CellSigFlow provides a biologically grounded representation of intercellular signaling. Unlike earlier cluster-level or pairwise methods, CellSigFlow resolves competition among ligands and receptors, enforces realistic upper and lower bounds on signaling flux, and captures multistep relay interactions, thereby offering a richer and more faithful characterization of cellular communication networks.

Through PDE-based simulations, we demonstrated that CellSigFlow outperforms established methods such as CellChat and COMMOT, consistently achieving higher concordance with ground-truth communication fields. This confirms the utility of our flow-based modeling in capturing the dynamic and competitive nature of signaling processes Application to the mouse olfactory bulb confirmed that CellFlow captures biologically coherent neuron–glia and synaptic signaling, driven by key axes such as Psap–Gpr37l1 and Ptn–Ptprz1. Together, these findings highlight CellSigFlow as a robust and scalable tool for single-cell and spatial transcriptomics analysis. By integrating spatial distance, molecular affinity, and receptor-ligand constraints into a unified optimization problem, CellSigFlow bridges the gap between theoretical modeling and real biological systems. Moving forward, this approach paves the way for systematically dissecting complex signaling landscapes across tissues and diseases, with the potential to uncover novel therapeutic insights and inform subsequent experimental validation.

**References:**

1. Jin S, Guerrero-Juarez CF, Zhang L, Chang I, Ramos R, Kuan CH, et al. Inference and analysis of cell–cell communication using CellChat. Nat Commun. 2021 Feb 17;12(1):1088.

2. Cang Z, Zhao Y, Almet AA, Stabell A, Ramos R, Plikus MV, et al. Screening cell–cell communication in spatial transcriptomics via collective optimal transport. Nat Methods. 2023 Feb;20(2):218–28.

